# Surgical Treatment and Clinical Outcomes of Petroclival Meningiomas: A Single-Center Experience of 107 Patients

**DOI:** 10.3389/fonc.2021.761284

**Published:** 2021-11-22

**Authors:** Baocheng Gao, Yongfa Zhang, Jiang Tan, Jinsong Ouyang, Bai Tai, Xianbao Cao, Tao Li, Shuang Hu

**Affiliations:** ^1^ Department of Neurosurgery, The First People’s Hospital of Yunnan Province, The Affiliated Hospital of Kunming University of Science and Technology, Kunming, China; ^2^ Department of Ear, Nose and Throat (ENT) and Head and Neck (HN) Surgery, The First People’s Hospital of Yunnan Province, The Affiliated Hospital of Kunming University of Science and Technology, Kunming, China

**Keywords:** petroclival, meningioma, surgical approach, skull base, neuronal function

## Abstract

**Objective:**

This study aimed to establish optimal surgical strategies *via* reviewing the clinical outcomes of various surgical approaches for the pertroclival meningiomas (PCMs).

**Methods:**

This retrospective study enrolled 107 patients with PCMs at the authors’ institution from year 2010 to 2020. Patient demographics, the clinical characteristics, various operative approaches, major morbidity, post-operative cranial nerve deficits and tumor progression or recurrence were analyzed.

**Results:**

The subtemporal transtentorial approach (STA), the Kawase approach (KA), the retrosigmoid approach (RSA) and the anterior sigmoid approach (ASA), namely the posterior petrosal approach (PPA) were adopted for 17 cases, 22 cases, 31 cases and 34 cases respectively. Total or subtotal resection was achieved in 96 cases (89.7%). The incidence of new-onset and aggravated cranial nerve dysfunction were 13.1% (14/107) and 10.4% (15/144), respectively. Furthermore, 14 cases suffered from intracranial infection, 9 cases had cerebrospinal fluid leakage, and 3 cases sustained intracranial hematoma (1 case underwent second operation). The mean preoperative and postoperative Karnofsky Performance Status (KPS) score was 80 (range 60-100) and 78.6 (range 0-100), but this was not statistically significant (*P*>0.05). After a mean follow-up of 5.1 years (range 0.3- 10.6 years), tumor progression or recurrence was confirmed in 23 cases. Two cases died from postoperative complications.

**Conclusions:**

For the treatment of PCMs, it is still a challenge to achieve total resection. With elaborate surgical plans and advanced microsurgical skills, most patients with PCMs can be rendered tumor resection with satisfactory extent and functional preservation, despite transient neurological deterioration during early postoperative periods.

## Introduction

Petroclival meningiomas (PCMs) refer to meningiomas that occur on the upper two-thirds of the clivus and medially to the internal auditory canal (IAC), adjacent to the major neurovascular structures, including brainstem, basilar artery, perforating arteries, and III-VII cranial nerves (CN) (1). Since most PCMs are World Health Organization (WHO) grade I tumors, the treatment goal should be curative total resection (TR) during the first operation when the arachnoid membranes are intact. In terms of the surgical approaches, the subtemporal transtentorial approach (STA) and the Kawase approach (KA) have the characteristics of short operative distance, convenient tumor base resection and less intraoperative bleeding but with the limitation for large posterior petrosal PCMS; the retrosigmoid approach (RSA) has the feature of fewer approach-related complications while the restriction to the petroclival region; the anterior sigmoid approach (ASA), also named the posterior petrosal approach (PPA), can facilitate the exposure of tumors, reduce the traction of the brainstem but perplexes the neurosurgeons for harder maneuver. Although there are many surgical approaches for PCMs and the relevant studies have repeatedly reported, the optimal choice for the operation is of extreme difficulty (2–5) and no uniform standard establishing the superiority of one approach over another is acknowledged currently (6, 7), due to the anatomical complexity, the multiformity of tumor invasion and the intricacy of a balance between neurofunctional preservation and tumor recurrence.

Despite remarkable advances in microsurgical techniques during the past decades, many recent reports (1) still reveal a low TR rate and suggest that aggressive extirpation is often associated with severe morbidity. For this reason, some authors proposed subtotal resection (STR) followed by radiotherapy in order to preserve the neurological functions. However, others insist that aggressive resection using various skull base surgical techniques should guarantee more favorable outcomes and the control of high-grade tumors. The surgical treatment of PCMs has always been a challenge for skull base neurosurgeons due to the deep location, complex adjacent structures and their scarcity (less than 0.15% of all intracranial meningiomas) (1). This study aimed to establish optimal surgical strategies *via* reviewing the clinical outcomes of various surgical approaches for PCMs based on our 107 cases.

## Materials and Methods

### Patients

The present study was approved by the Ethics Committee of the First People’s Hospital of Yunnan Province. All procedures performed in studies that involved human participants were in accordance with the ethics standards of the Institutional and National Research Committee, and the 1964 Helsinki Declaration and its later amendments or comparable ethics standards. Written consent was obtained from the patients.

This retrospective study enrolled 107 patients from the Department of Neurosurgery, the First People’s Hospital of Yunnan Province, from January 2010 and December 2020. All patients were diagnosed with PCMs based on radiological and histopathological results. The exclusion criteria were as follows: (1) patients with a magnetic resonance imaging (MRI) revealing that the main body of the tumor is located on the inferior clivus or lateral wall of the IAC; (2) patients with multiple (≥2) intracranial meningiomas; (3) patients with no successful follow-up.

### Clinical and Radiological Evaluation

The demographic and clinical profiles were collected, and the operative logs were reviewed. The neurological functions were evaluated preoperatively and 2 weeks after operation using the Karnofsky Performance Status (KPS) scale, and all patients underwent perioperative MRI scans. The extent of the resection was determined based on the intraoperative finding and enhanced MRI within 72 hours postoperatively. Total resection was defined as Simpson grade I and II, subtotal resection was defined as Simpson grade III and IV, and partial resection was defined as Simpson grade V. According to the classification system proposed by Kawase et al. (8) in 1996 and Ichimura et al. (9) in 2008, the PCMs were divided into four groups: upper clival type, cavernous type, tentorium type, and petrous apex type. According to the size-based classification criteria proposed by Sekhar et al. (10), measured on the maximum diameter, tumors were small (<10 mm), medium (10-24 mm), large (25-44 mm), and giant (≥45 mm).

### Surgical Treatment

The surgical resection was performed with the assistance of electrophysiological monitoring, and the selection of surgical approaches was shown in **Table 1**. For petrous apex type PCMs, the subtemporal transtentorium approach (STA) was preferred. However, when the tumor was large, the Kawase approach (KA) was used. For cavernous type PCMs, KA was the first choice. However, when the tumor was located in the posterior fossa and barely invaded the middle fossa, the retrosigmoid approach (RSA) was used. For upper clivus type PCMs, the anterior sigmoid sinus approach (ASA) was adopted. However, when the tumor did not cross the midline of the clivus, the KA or RSA were selected. For tentorium type PCMs, the STA was used when the tumor size was small or medium, while the tumor was large or giant the RSA or ASA should be used depending on whether the tumor crossed the midline of the clivus.

**Table 1:** Selection of surgical approaches based on imaging classification.

Imaging classification	Cases (*n*)	Approach (*n*)					TR (*n*)	SR (*n*)	PR (*n*)
		STA	KA	RSA	ASA	CA			
PAT	16	11	5	0	0	0	16	0	0
TT	38	6	0	19	13	0	15	23	0
CT	19	0	13	5	0	1^a^	0	10	9
UCT	34	0	4	7	21	2^b^	26	6	2
SUM	107	17	22	31	34	3	57	39	11

PAT, petrous apex type; TT, tentorium type; CT, Cavernous type; UCT, upper clivus type; SUM, summation; STA, subtemporal transtentorium approach; KA, Kawase approach; RSA, retrosigmoid approach; ASA, anterior sigmoid approach; CA, combined posterior and anterior petrosal approach; TR, total resection; SR, subtotal resection; PR, partial resection.

a The Fisch’s type A approach was used, because the tumor invaded the infratemporal fossa.

b The combined posterior and anterior petrosal approach was used, because the tumors extensively invaded the cavernous sinus and crossed the midline of the clivus.

### Follow-Up and Statistical Analysis

The follow-up was implemented on an outpatient basis. Clinical and radiological examinations were performed. The SPSS 20.0 software (IBM Corp., Armonk, NY, USA) was used for the statistical analysis. The KPS scores was compared using *t*-test, and the progression or recurrence rate was evaluated using a chi-square test (or a Fisher exact test when necessary). And the probability (*P*) values <0.05 were considered statistically significant.

## Results

### Demographic Characteristics

There were 28 males (26.2%) and 79 females (73.8%), with an average age of 42.8 years old (range 19-72 years). The clinical symptoms were as follows: headache in 33 cases (30.8%); CN III, IV, and/or VI dysfunction (diplopia) in 25 cases (23.4%); CN V dysfunction (trigeminal neuralgia and/or facial numbness) in 61 cases (57.0%); CN VIII dysfunction (hearing impairment) in 28 cases (26.2%), CN VII dysfunction (facial paralysis) in 11 cases (10.3%); posterior group cranial nerves dysfunction in 19 cases (17.8%); ataxia in 24 cases (22.4%), and progressive hemiparesis in 10 cases (9.3%). In addition, 13 patients (12.1%) were asymptomatic, who requested surgical operation due to psychological stress and other factors. The median duration between onset and surgical treatment were 29 months (range 4-156 months). The mean tumor size was 39.1 mm (range 6.2-75.9 mm), in which 59 patients (55.1%) had large tumors, 40 cases (37.4%) with giant tumors, 6 cases (5.6%) had medium tumors, and only 2 patients (1.9%) had small tumors. The mean follow-up period was 5.1 years (range 0.3-10.6 years). The patient characteristics and detailed clinical information are shown in **Table 2**.

**Table 2:** Demographic data for 107 patients with petroclival meningiomas.

Demographic data	Value
Mean age (range, yrs)	42.8 (19-72)
Male/female ratio	28:79
Mean preoperative KPS score (range)	80 (60-100)
Mean clinical follow-up (range, yrs)	5.1 (0.3-10.6)
Symptoms & signs at onset (no., %)	
Headache	33 (30.8%)
Diplopia	25 (23.4%)
Trigeminal neuralgia and/or facial numbness	61 (57.0%)
Acoustic-facial bundle dysfunction	39 (36.4%)
Posterior cranial nerve dysfunction	19 (17.8%)
Ataxia	24 (22.4%)
Progressive hemiparesis	10 (9.3%)
Asymptomatic and others	13 (12.1%)
Mean tumor size (range, mm)	39.1 (6.2-75.9)
Small (<10 mm)	2 (1.9%)
Medium (10mm≤diameter<25mm)	6 (5.6%)
Large (25mm≤diameter<45mm)	59 (55.1%)
Giant (≥45mm)	40 (37.4%)

### Surgical Approaches and the Extent of Surgical Resection

**Figure 1:**
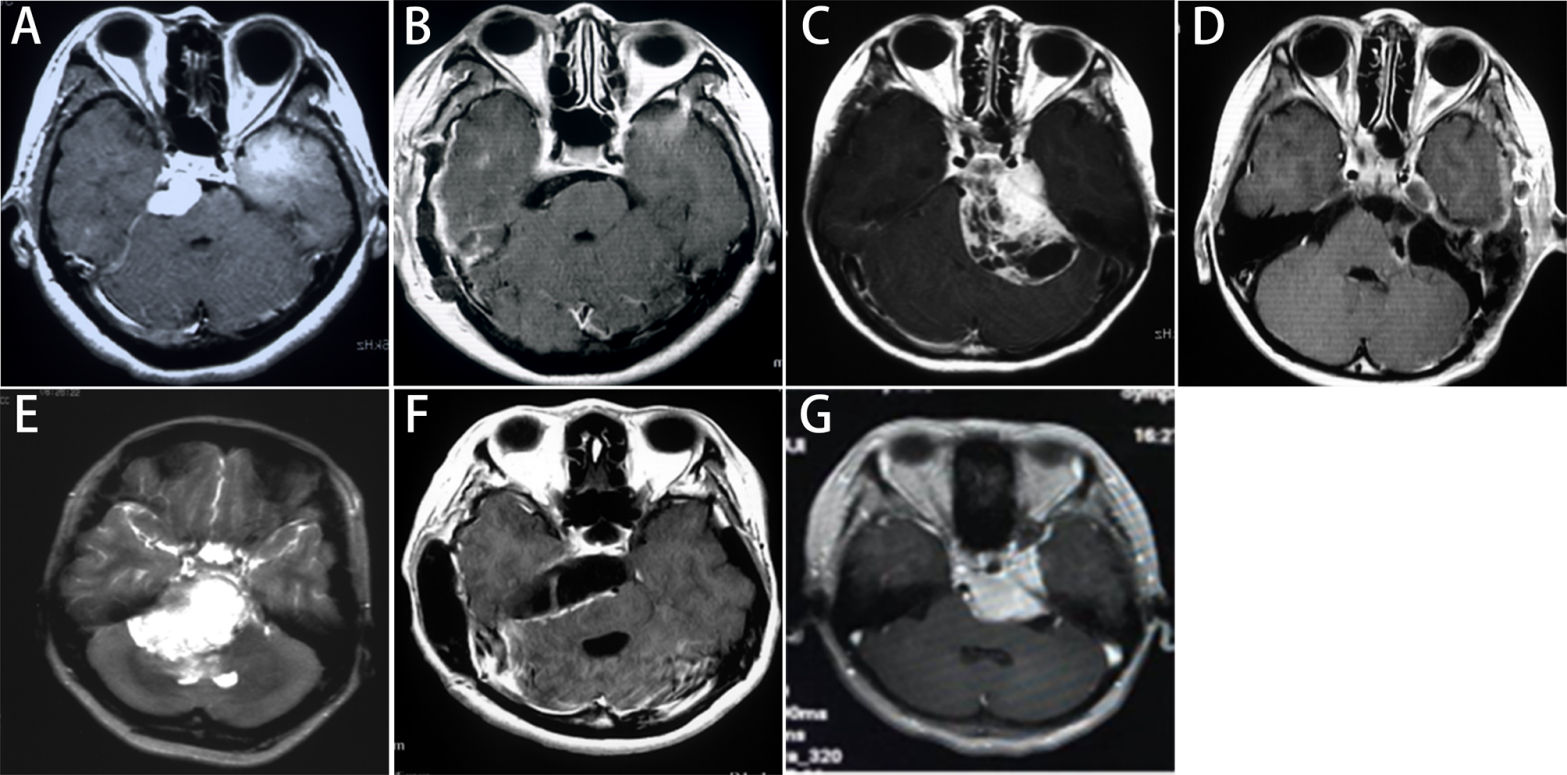
Imaging classification and selection of surgical approaches for PCMs. Petrous apex type: **(A)** The preoperative enhanced MRI shows that the subtemporal transtentorial approach was used; **(B)** The MRI within postoperative 72 hours shows a Simpson grade I resection. Tentorium type: **(C)** The preoperative enhanced MRI shows that the retrosigmoid approach was used; **(D)** The MRI within postoperative 72 hours shows that the tumor invading the posterior wall of the cavernous sinus was removed, yielding a Simpson grade II resection. Upper clivus type: **(E)** The preoperative enhanced MRI shows that the anterior sigmoid approach was used; **(F)** The MRI within postoperative 72 hours shows a Simpson grade II resection. Cavernous type: **(G)** The preoperative enhanced MRI shows that the Kawase approach was used, and a Simpson grade III resection was achieved.

STA was used in 17 cases, KA was selected in 22 cases, ASA was adopted in 34 cases, and RSA was employed in 31 cases. The combined posterior and anterior petrosal approach was used in two cases and the Fisch’s type A approach was used in one case. A total of 57 patients underwent Simpson grade I or II resection (total resection, **Figure 1**). Total or subtotal resection was achieved in 96 patients (89.7%), whereas subtotal and partial resection were achieved in 39 patients and 11 patients respectively. Details of the surgical approaches and the extent of removal were shown in **Table 1**.

### Postoperative Histopathology of the Tumors

**Figure 2:**
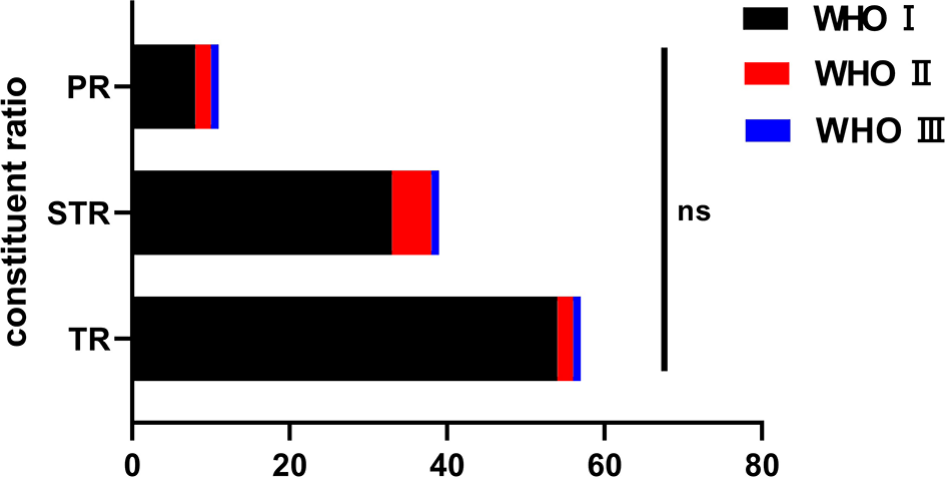
The pathological classification among the groups and the resection degree. ns, no significance.

Pathology was reported as WHO grade I in 95 cases (88.8%), 9 tumors were reported as WHO grade II (atypical type) and 3 as WHO grade III (anaplastic type). Among the 95 cases of grade I meningiomas, the meningothelial subtype (66 cases, 69.5%) was most common, followed by transitional subtype (17 patients, 17.9%), secretory subtypes (6 cases, 6.3%) and other subtypes (6 cases, 6.3%). There was no statistical significance between the WHO grade and degree of tumor resection (P>0.05, **Figure 2**).

### Postoperative Complications and Functional Outcome

**Figure 3:**
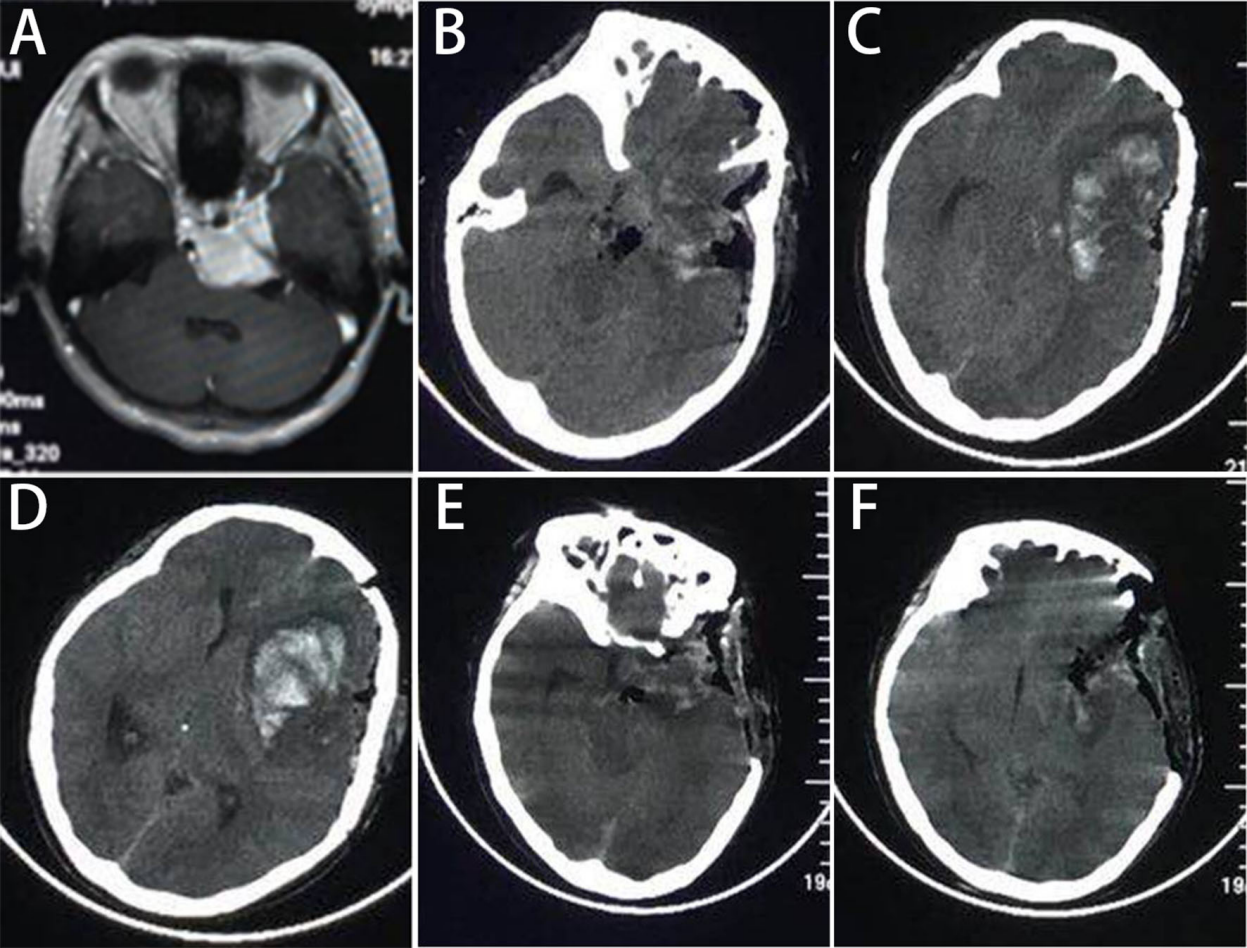
The one patient who had postoperative hematoma and needed second operation. **(A)** The MR scan shows the cavernous type PCMs, and **(B‒D)** the CT at 6h postoperatively show a hematoma in the frontal lobe with midline displacement. **(E, F)** The postoperative CT shows that the hematoma was evacuated.

The incidence of new-onset and aggravated cranial nerve dysfunction were 13.1% (14/107) and 10.4% (15/144), respectively. The CN IV to VIII dysfunction was common, and most of them had improved at recent follow-up. Detailed cranial nerve dysfunctions were shown in **Table 3**. Other major postoperative morbidities were intracranial infection (14 cases, 13.1%), cerebrospinal fluid leakage (9 patients, 8.4%), postoperative hematoma (3 patients, 2.8%), in which 1 case needed second operation (**Figure 3**). Two patients with postoperative hematoma died of pneumonia and multiple organ failure, respectively. The mean preoperative and postoperative KPS scores were 80 (range 60-100) and 78.6 (range 0-100), respectively. This was not statistically significant (*t*=-0.102, *P*=0.922). Furthermore, at their most recent follow-up, 57 cases (53.3%) were stable with no worsened KPS, and 36 cases (33.6%) had improved, only 14 patients (13.1%) had aggravated KPS score.

**Table 3:** Dysfunctions of the cranial nerves.

Cranial nerve	Preoperation	2 weeks after operation					Follow-up	
		Unchanged	Aggravated	New-onset	Improved			
III	9	4	1	1	4		5	
IV	4	1	2	3	1		3	
V	61	17	2	2	42		11	
VI	12	6	1	1	5		5	
VII	11	3	5	4	3		6	
VIII	28	13	3	2	12		15	
IX-XII	19	7	1	1	11		2	
Sum	144	51	15	14	78		47	

Sum, summation.

### Tumor Progression or Recurrence

**Figure 4:**
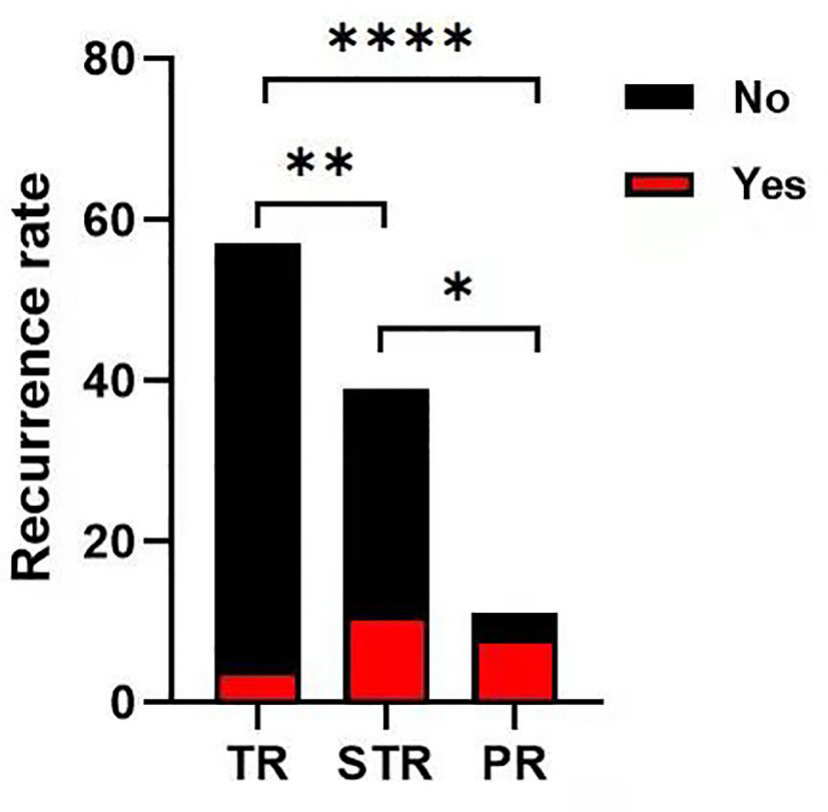
The progression or recurrence rate among the groups. The progression or recurrence rate was 72.7% (8 of 11 cases) in PR group, 28.2% (11 of 39 cases) in STR group, 7% (4 of 57 cases) in TR group, respectively. The differences were statistically significant between each two groups (Chi-square test, *p < 0.05 **p < 0.01, ****p < 0.0001).

**Figure 5:**
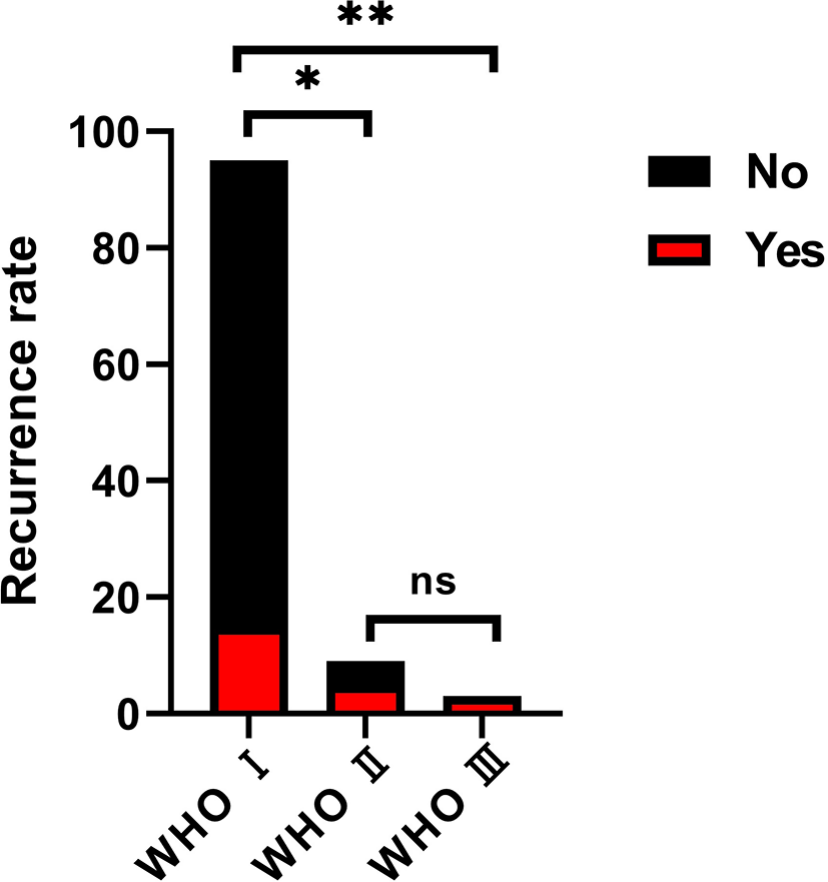
The progression or recurrence rate among groups on the basis of the pathological classification. the progression or recurrence rate was 15.8% (15/95 cases), 55.6% (5/9 patients) and 100% in the WHO grade I, II and III group, respectively. This was statistically significant when WHO grade I compared with WHO grade II (Chi-square test, *p < 0.05) and III (Chi-square test, **p < 0.05), but no significance between grade II and grade III. (Chi-square test, ns, no significance).

During the follow-up, overall progression or recurrence was confirmed in 23 cases (21.5%). According to the extent of tumor resection, the progression or recurrence rate was 72.7% (8 of 11 patients) in the partial resection group, 28.2% (11 of 39 cases) in the subtotal resection group, and 7% (4 of 57 patients) in the total resection group. This was statistically significant among groups (P<0.05, **Figure 4**). According to the pathological subtypes, the progression or recurrence rate was 15.8% (15/95 cases), 55.6% (5/9 patients) and 100% in the WHO grade I, II and III group, respectively. This was statistically significant when WHO grade I compared with WHO grade II and III (P<0.05, **Figure 5**).

## Discussion

PCMs usually adjoin the brainstem and cranial nerves. Although the surgical techniques have been greatly improved in recent years, the total resection rate of PCMs remains low (approximately 30-70%), and the incidence of postoperative complications is approximately 20-30% (11). Hence surgical management is still one of the most challenging problems for skull base neurosurgeons (12–15), and the selection of surgical approaches for PCMs has become a hot topic (3, 16, 17). According to the experience of the investigators, petrous apex type PCMs are often located above the trigeminal nerve, which is often displaced by the tumor. Therefore, STA gives priority to surgeons to cut off the tumor base and effectively reduces bleeding. In the study, a total of 17 patients (11 cases of petrous apex type and 6 cases of tentorial type) underwent surgical resection *via* the STA. It was found that this approach could facilitate the exposure and complete removal of small-to-medium petrous apex type and tentorial type PCMs. Cavernous type PCMs often invade the middle and posterior fossa in a dumbbell-like fashion. The KA can help anteriorly reach the anterior clinoid process (18) and posteriorly reach the plane of the IAC, completely expose the lateral wall of the cavernous sinus, which facilitates the gross total resection. In the present study, a total of 22 cases (13 cases of cavernous type, 5 cases of petrous apex type, and 4 cases of upper clivus type) were treated with the KA. The investigators consider the KA to be suitable for the majority of PCMs, especially the cavernous type tumors that straddle the middle and posterior fossa. Upper clivus type PCMs often invade across the midline of the clivus, and the ASA can reduce the traction of the brainstem (19). In this study, a total of 34 patients (21 patients with upper clivus type PCM and 13 patients with tentorium type PCM) were treated with the ASA. The ASA is preferred for large tumors, especially those involving the lateral part of the IAC and midline of the clivus (20). For tentorium type PCMs, the tumor base is located at the tentorium, and the tumor often grows into the posterior cranial fossa. When the tumor was large to giant, surgical exposure *via* the STA is limited, whereas the RSA can serve the tumor exposure. In addition, through the RSA, the separation of the tumor from the trigeminal nerve and acoustic and facial nerve was under direct vision, which can effectively reduce the incidence of postoperative complications. Moreover, the tentorium can be incised and the tumor that invades the posterior wall of the cavernous sinus can also be well-exposed. A total of 31 cases (19 cases of tentorium type, 7 cases of upper clivus type, and 5 cases of cavernous type) were treated using the RSA.

In addition, the selection of a specific surgical approach should be based on the location of the tumor base, tumor size, degree of invasion, and surgeon’s familiarity with the approach. If the tumor extensively involved the cavernous sinus and midline of the clivus, the combined posterior and anterior petrosal approach should be applied. In our study, two patients were treated with this combined approach. Additionally, one patient underwent the Fisch A-type approach due to the tumor invasion into the infratemporal fossa. Moreover, preoperative assessment of the venous anatomy for surgical planning is also essential (21, 22). We recommend that all patients with PCMs should have MRV or CTV imaging, or/and DSA when it is necessary, before surgery. For example, if the preoperative results show that the Labbé vein flows into the superior petrosal sinus or the patient have a high jugular bulb, the lateral skull base approach (such as ASA) should be avoided prudently. If the tumor invades the middle and posterior fossa, the modified ASA approach is used to protect the superior petrosal sinus (23, 24). Likewise, if the Labbé vein drains into the transverse sinus at the anterior part of the temporal lobe, the STA or KA approach is restricted. If the tumor is small and slightly invades the cavernous sinus, we can also use STA or KA. In order to avoid damage to the Labbé vein, we often use mannitol or implant the lumbar cistern drainage to lower intracranial pressure. In the meantime, according to the situation of the Labbé vein during the operation, sharp separation or the removal of part of temporal lobe can be adopted to increase its mobility.

In this study, total or subtotal resection was achieved in 96 cases (89.7%). For petrous apex type PCMs, 16 cases (100%) had total resection. For upper clivus type PCMs, total resection was achieved in 26 cases (76.5%). For tentorium type PCMs, 15 cases achieved total resection, and 23 cases (60.5%) subtotal resection. Cavernous type PCMs was characterized as the tight adhesion between the tumor and adjacent nerves and vessels in the cavernous sinus. Ten cases (52.6%) were achieved subtotal resection and 9 cases (47.4%) merely partial resection.

Another difficulty in the surgical management of PCMs is the intraoperative protection of cranial nerves (25, 26). A most frequent complication for any skull base approach is the ever-present risk of the injury to the CNs. According to the literature, the incidence of cranial nerve dysfunction after surgery is 20-100% (27–29). In this study, preoperative cranial nerve dysfunction mainly involved the III-IX cranial nerves. The incidence of new-onset and aggravated cranial nerve dysfunction were 13.1% (14/107) and 10.4% (15/144), respectively. Most neurological disorders were improved during the follow-up. For intraoperative neurological protection, the experience of the investigators was as follows: (1) The trigeminal nerve is located below the superior petrosal sinus, thus the cauterization of superior petrosal sinus should be given with more attention. The trochlear nerve is often located in the medial of the tumor, and the facial nerve and vestibular nerve are located on the lower lateral side of the tumor, the separation of them from the tumor should along the arachnoid membrane interface hence. (2) If the cranial nerves were tightly enclosed, such as III-VI nerves in cavernous type PCMs, the cranial nerve dysfunction is usually aggravated postoperatively. Therefore, the goal of surgery has been transferred from the total resection to maximum preservation, since preserving neurological functions is pivotal to improve postoperative quality of life. Thus, we recommend incomplete resection followed by adjuvant radiotherapy for this type PCMs (3). Serviceable hearing preservation is also very important. The hearing protection during tumor resection is mainly the protection of the auditory nerve. The same as for vestibular schwannomas, the translabyrinthine approach (labyrinthectomy) sacrifices hearing to achieve greater exposure and total resection, whereas the middle fossa approach (such as STA and KA) and retrosigmoid approach offer the possibility of hearing preservation (30). This highly influences the choice of surgical approaches: if PCM patients have practical hearing before surgery, the trans-middle cranial fossa approaches and retrosigmoid sinus approach can be used; if the patient does not have practical hearing before surgery, the translabyrinthine approach may be considered based on the tumor location. However, for PCMs, patients often suffer from cranial nerve dysfunction in CN V and posterior group cranial nerves; the vestibulocochlear nerve complex often located caudally, making it a crucial maneuver to keep an intact arachnoid plane between the tumor and the surrounding structures. Under the protection of electrophysiological testing, the in-capsule tumor decompression should be implemented, and then the sharp separation between the residual envelope from the surrounding structures upon the arachnoid interface. The complete arachnoid interface must be ensured, so that the maximum tumor resection and hearing preservation can be achieved. In this study, most patients with preoperative hearing impairment had an improvement significantly at follow-up. Therefore, we claim that meticulous techniques and the knowledge of microsurgical anatomy shall lead to feasible hearing preservation with maximum tumor removal under contemporary circumstances.

With respect to the other postoperative complications, there were 14 cases suffering intracranial infection, 9 cases had cerebrospinal fluid leakage, and 3 cases with postoperative hematoma (1 case needed second operation). And there were two deaths because of pneumonia and multiple organ failure after postoperative hematoma. It is clear that modern cranial base techniques and resection skills can significantly reduce the complications. Despite transient neurological deterioration that may occurred during early postoperative periods after total resection. In this group, the incidence of new-onset and aggravated cranial nerve dysfunction were 13.1% (14/107) and 10.4% (15/144), respectively. Though the mean preoperative and postoperative KPS scores were 80 (range 60-100 points) and 78.6 (range 0-100 points) respectively, this was not statistically significant (*t*=-0.102, *P*=0.922). Furthermore, at their most recent follow-up, 57 cases (53.3%) were stable with no worsened KPS, 36 cases (33.6%) had improved, only 14 patients (13.1%) had aggravated KPS score. In addition, the progression or recurrence rate was statistically significant among TR, STR and PR groups (P<0.05) and there was no statistically significance between the WHO grade and degree of tumor resection (P<0.05). Thus, we suggest total resection appears to be advantageous for various skull base approaches on PCMs. This is consistent with Almefty et al. (1) who concluded that multiple skull base approaches to PCMs not only facilitate an improved chance of total resection, but also decrease the risk of morbidity. In our study, there was a statistically significance of the progression or recurrence rate when WHO grade I compared with WHO grade II and III, but it was insignificant between WHO grade II and III, which might be due to the invasive nature of grade II and III tumors, or simply the bias caused by the small amount of the two groups and the shortness of follow-up time.

## Conclusions

In conclusion, resection of PCMs remains a challenge. The optimal surgical approach depends on the size, extension of the tumor and the anatomical relationship between the tumor and the cranial nerves. RSA and petrosal approaches were the most commonly used. With elaborate surgical plans and advanced microsurgical skills, most patients with PCMs can be rendered tumor resection with satisfactory extent and functional preservation, despite transient neurological deterioration during early postoperative periods.

## Data Availability Statement

The original contributions presented in the study are included in the article/supplementary material. Further inquiries can be directed to the corresponding authors.

## Ethics Statement

The studies involving human participants were reviewed and approved by the Ethics Committee of the First People’s Hospital of Yunnan Province. The patients/participants provided their written informed consent to participate in this study.

## Author Contributions

BG initiated the study and wrote the manuscript. YZ and JT collected the data. JO, BT, and XC analyzed the data. TL and SH coordinated the study and revised the article. All authors contributed to the article and approved the submitted version.

## Funding

This work was supported by National Natural Science Foundation of China (Grant NO. 82001278), The Fund for Young Doctors with the First People’s Hospital of Yunnan Province (Grant NO. KHBS-2020-014), Yunnan Fundamental Research Projects (Grant NO. 202101AU070106), and Joint Projects of Yunnan Provincial Science and Technology Department and Kunming Medical University for Applied Basic Research (Grant NO. 202101AY070001-252).

## Conflict of Interest

The authors declare that the research was conducted in the absence of any commercial or financial relationships that could be construed as a potential conflict of interest.

## Publisher’s Note

All claims expressed in this article are solely those of the authors and do not necessarily represent those of their affiliated organizations, or those of the publisher, the editors and the reviewers. Any product that may be evaluated in this article, or claim that may be made by its manufacturer, is not guaranteed or endorsed by the publisher.
